# Multiplication rate variation of malaria parasites from hospital cases and community infections

**DOI:** 10.1038/s41598-024-82916-4

**Published:** 2025-01-03

**Authors:** Lindsay B. Stewart, Elena Lantero Escolar, James Philpott, Antoine Claessens, Alfred Amambua-Ngwa, David J. Conway

**Affiliations:** 1 Department of Infection Biology, London School of Hygiene and Tropical Medicine, Keppel St, London, WC1E 7HT UK; 2 LPHI, MIVEGEC, INSERM, CNRS, IRD, University of Montpellier, Montpellier, France; 3 MRC Unit The Gambia at London School of Hygiene and Tropical Medicine, Banjul, The Gambia; 4 Department of Infection Biology, Faculty of Infectious and Tropical Diseases, London School of Hygiene and Tropical Medicine, Keppel St, London, WC1E 7HT UK

**Keywords:** Malaria, Virulence, Epidemiology, Parasite multiplication, Hospital, Community, Infection, Parasite biology

## Abstract

The significance of multiplication rate variation in malaria parasites needs to be determined, particularly for *Plasmodium falciparum*, the species that causes most virulent infections. To investigate this, parasites from cases presenting to hospital in The Gambia and from local community infections were culture-established and then tested under exponential growth conditions in a standardised six-day multiplication rate assay. The multiplication rate distribution was lower than seen previously in clinical isolates from another area in West Africa where infection is more highly endemic. Multiplication rates were higher in cultured isolates derived from hospital cases (*N* = 23, mean = 2.9-fold per 48 h) than in those from community infections (*N* = 11, mean = 1.8-fold)(Mann-Whitney *P* < 0.001). There was a positive correlation between levels of parasitaemia in peripheral blood of sampled individuals and multiplication rates of the isolates in culture (Spearman’s rho = 0.45, *P* = 0.017). There was no significant difference between isolates containing single parasite genotypes or multiple genotypes at the time of assay, suggesting that parasites do not modify their multiplication rates in response to the presence of different genotypes. It will be important to uncover the mechanisms of this intrinsic multiplication rate variation, and to also investigate the epidemiological distribution and potential associations with infection phenotypes in other populations.

**Supplementary Information:**

The online version contains supplementary material available at 10.1038/s41598-024-82916-4.

## Introduction

All clinical malaria is caused by asexually replicating blood-stage parasites, with high infection loads leading to severe disease in some cases^1,2^. Despite previous advances in malaria control, the global burden of disease remains very high and extremely inequitable, more than half of all annual malaria cases and deaths being due to *Plasmodium falciparum* in West Africa^3^. Within this most important parasite species, any naturally occurring variation in multiplication potential would be likely to influence infection load and disease risk, so its significance needs to be determined.

Parasite multiplication rates cannot normally be measured directly in patients, as therapy requires prompt anti-parasite treatment, although modelling of multiple parameters in clinical samples suggests there is intrinsic variation of parasite multiplication potential^2,4^. It is difficult to estimate parasite multiplication rates in a standardised manner in induced human experimental infections with laboratory strains of *P. falciparum*^5^. One such in vivo study suggested a multiplication rate difference between two unrelated *P. falciparum* laboratory strains^6^, although another independent study did not indicate a difference after a similar comparison^7^. Interpretations from experimental infections are also complicated by genomic changes occurring within laboratory strains which are not seen in natural infections^8^.

Studies of parasites in short-term cultured clinical isolates have indicated variation in multiplication rates which would not be affected by culture-acquired mutants. Measuring *P. falciparum* multiplication in the first cycle ex vivo, studies in Thailand and Uganda^9,10^showed higher rates in severe malaria compared to mild malaria isolates, although a study of parasites from Mali and Kenya indicated no difference between severe and mild malaria isolates^11^. Although interesting, a limitation of these comparisons is that initial viability of parasites within clinical samples is generally variable, affecting interpretations of multiplication in the first ex vivo cycle.

Malaria parasite multiplication rate measurements can be more controlled under conditions of continuous in vitro culture. After establishment in culture for a few weeks, testing under exponential growth conditions has shown multiplication rates of *P. falciparum* in diverse clinical isolates to have a range from approximately 2-fold to 8-fold per 48-hour period corresponding to a typical asexual cycle time^12,13^. Interestingly, among isolates from a highly endemic population in Ghana, there was a significantly positive correlation between exponential multiplication rates in culture and the levels of parasitaemia seen in the peripheral blood samples^13^, suggesting that in vitro assay measurement has some correspondence with multiplication potential in vivo. Such multiplication rate variation among newly established clinical isolates is not affected by parasite mutants that may become common in longer-term cultures, which affect the most widely cultured laboratory-adapted strains of *P. falciparum*^8,14^.

To investigate naturally occurring parasite multiplication rate variation, it is important not only to study isolates from hospital clinical cases, but also from infections within communities, as some of these might contain parasites with lower intrinsic multiplication rates than those that cause clinical malaria. Although such community infections are common, it is generally harder to study parasites from these as they often have very low levels of parasitaemia^15^. Here, samples were analysed from The Gambia, where *P. falciparum* remains continuously endemic although incidence has declined over the past decades^16–20^. To analyse intrinsic multiplication rates of parasites, isolates from blood samples of hospital cases and community infections were established and assayed under exponential growth conditions in culture. This has enabled the first ever comparison of multiplication rates of malaria parasites from hospital cases and from community infections, revealing significant differences.

## Results

### Multiplication rates in cultured isolates from hospital and community samples

**Fig. 1:**
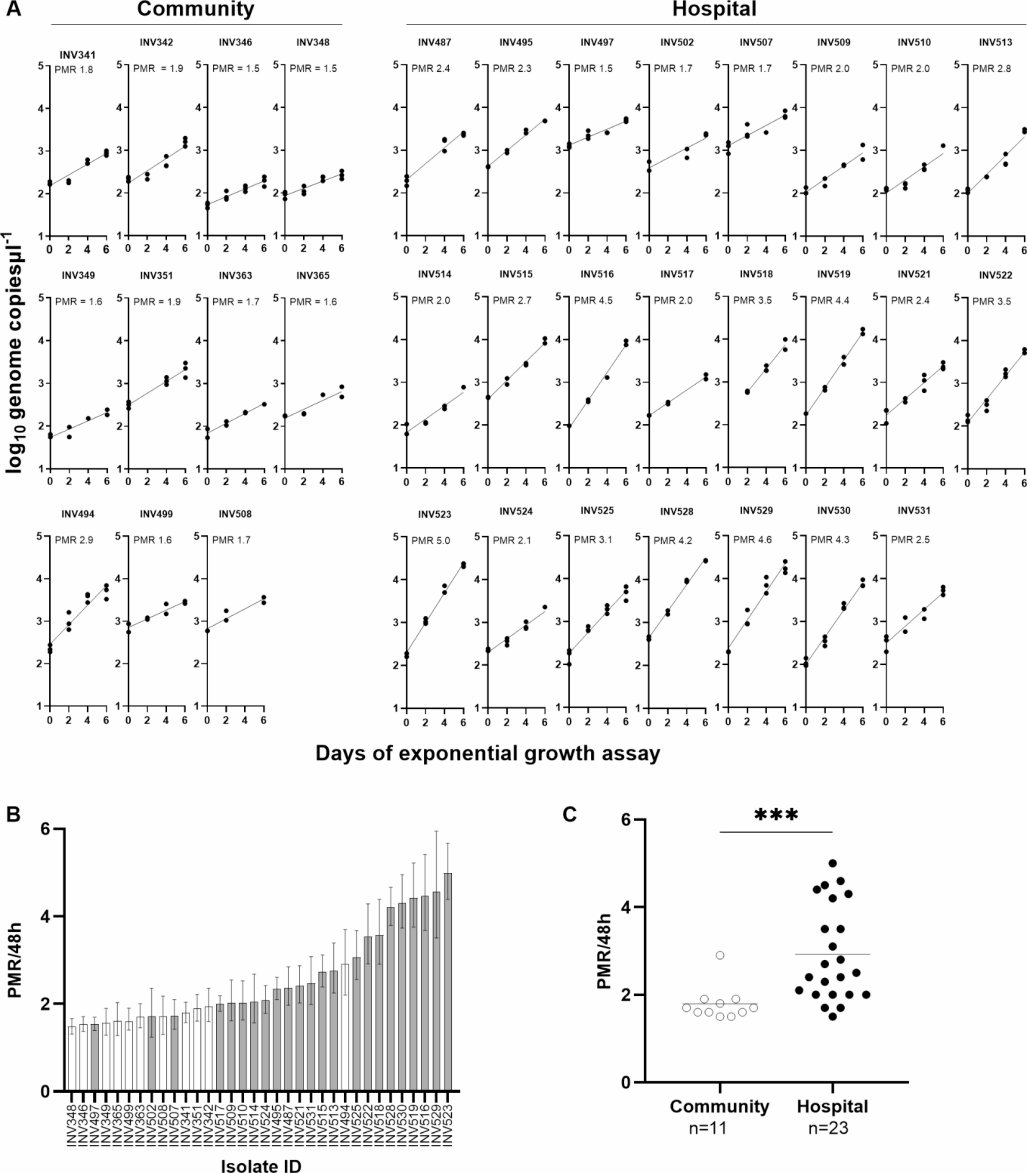
**(A)** Exponential multiplication rate assays of 34 Gambian *P. falciparum* isolates. Community-based isolates (*N* = 11) are shown on the left, and hospital-based isolates (*N* = 23) on the right. After at least 3 weeks of continuous culture as detailed in the Methods section, each isolate was tested in a 6-day exponential growth assay conducted in triplicate with erythrocytes from three different donors. The plots show parasite genome copies (Log_10_ scale) per microlitre of DNA extracted from each culture timepoint (each culture sample was extracted into a 50 µl volume of DNA). The overall parasite multiplication rate (PMR) for each isolate is derived from all of the timepoint data in all of the triplicates over the six-day period, applying a linear model on the Log10 density data, and this is expressed in non-logarithmic form as the multiplicative fold-change per nominal unit time of 48 h. Other details and numerical data for each isolate are shown in Supplementary Tables S1 and S2. **(B)** Bar plot showing the multiplication rate (with 95% confidence intervals) of each isolate, ranked from the lowest (1.5-fold) to the highest (5.0-fold). Community-based isolates (shown with white bars) are skewed towards the left of the distribution, with generally lower multiplication rates than the hospital-based isolates (shaded bars). **(C)** The distributions of parasite multiplication rates in community-based isolates (mean = 1.8-fold) and hospital-based isolates (mean = 2.9-fold) are significantly different (Mann-Whitney test, *P* < 0.001).

Cryopreserved blood samples from malaria cases presenting at Basse Hospital and local community *P. falciparum* infections were thawed and cultured under the same conditions for at least 20 days prior to testing in six-day exponential multiplication rate assays, yielding high quality data for 34 isolates (Fig. 1A, Supplementary Tables S1 and S2). Community-based isolates (*N* = 11) showed a low range of multiplication rates from 1.5-fold to 2.9-fold (expressed as the fold-change per 48 h corresponding to a typical cycle length). The hospital-based isolates (*N* = 23) showed a range from 1.5-fold to 5.0-fold.

Analysis of the ranked parasite multiplication rates shows that community-based isolates are skewed towards the low end of the distribution (Fig. 1B). The distributions of multiplication rates in community-based isolates (mean = 1.8-fold) and hospital-based isolates (mean = 2.9-fold) are significantly different (Mann Whitney test, *P* < 0.001) (Fig. 1C).

Interestingly, although these Gambian hospital-based isolates had higher multiplication rates than the community-based isolates, they were lower than previously seen for clinical isolates sampled from a more highly endemic area in Ghana (mean = 4.3-fold with a range from 2.0-fold to 8.0-fold, tested after 25 days in culture)^13^. The Gambian patients had generally lower levels of parasitaemia (Supplementary Table S1) compared with the previously studied Ghanaian patients. Peripheral blood parasitaemia levels in the patient samples from Ghana had previously been shown to correlate positively with parasite multiplication rates of the isolates in culture^13^.

## Peripheral blood parasitaemia and age of subjects

**Fig. 2:**
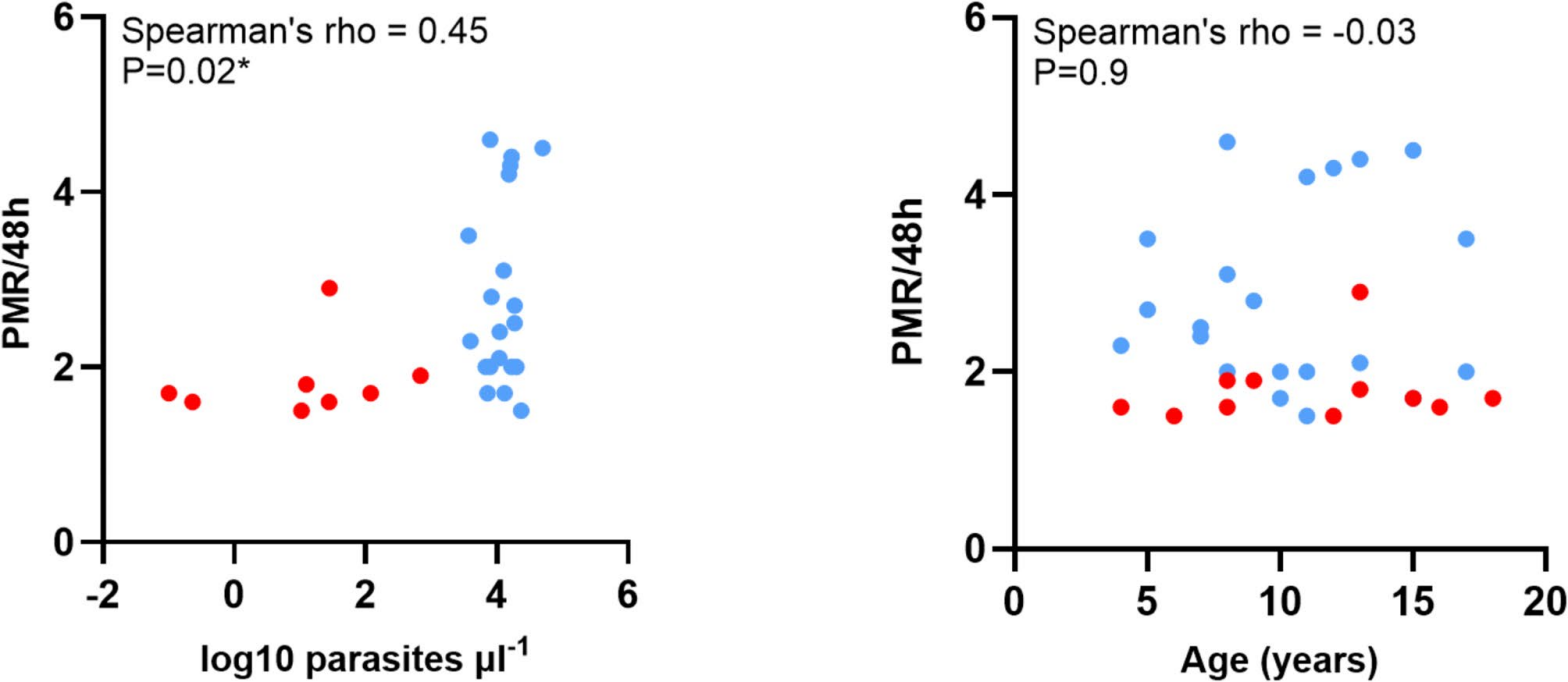
**(A)** Correlation between parasite multiplication rates under exponential growth conditions in culture and the parasitaemia measured in Gambian individuals at time of original blood sampling. Red symbols show community isolates, and blue symbols show hospital isolates (measurements were available for all except six of the 34 isolates as shown in Supplementary Table S1). Analysis of all samples together shows a significant positive correlation (*N* = 28, Spearman’s rho = 0.45, *P* = 0.017). Although there is limited power to test correlations within each of the subgroups, these have slight positive trends (community isolates *N* = 8, rho = 0.54; hospital isolates *N* = 20, rho = 0.06). **(B)** There is no significant correlation between parasite multiplication rates and age of patients (available for all except two of the isolates, *N* = 32, rho = − 0.03, *P* = 0.9).

The estimated level of peripheral blood parasitaemia in the Gambian study subjects (Supplementary Table S1), was tested for correlation with parasite multiplication rates in the culture assays. Analysis of all Gambian samples shows a highly significant positive correlation (Spearman’s rho = 0.45, *P* = 0.017) (Fig. 2A). There were positive trends in each of the hospital and community subgroups, but multiple hypothesis testing was avoided given absence of separate a priori hypotheses and lower numbers in each.

In contrast, there was no significant correlation between parasite multiplication rates and age of study subjects in The Gambia (Spearman’s rho = − 0.03, *P* = 0.9) (Fig. 2B). This is consistent with previous results from Ghanaian clinical isolates which showed that parasite multiplication rates were not correlated with the age of patients^13^.

## Genotypic mixedness and presence of gametocytes

**Fig. 3:**
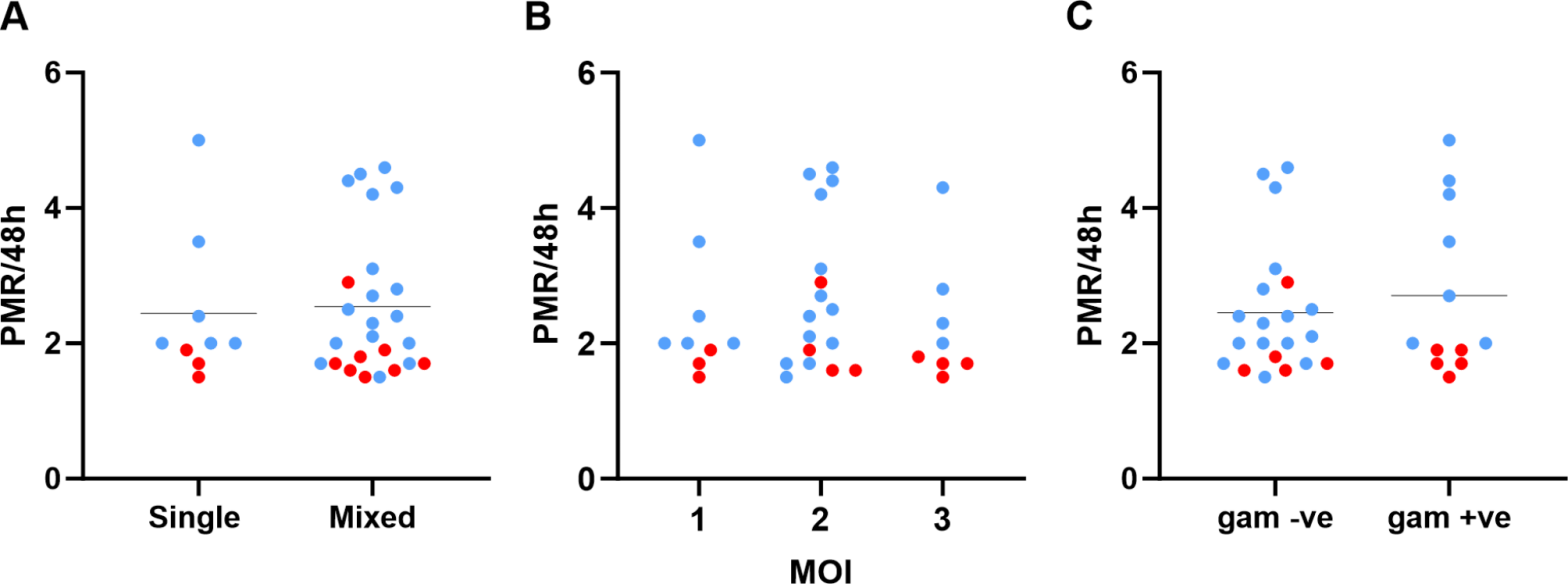
Multiplication rates of Gambian *P. falciparum* isolates under exponential growth conditions are not associated with the numbers of genotypes detected in each isolate or with the detected presence of gametocytes in culture at the time of assay. Red symbols show community isolates, and blue symbols show hospital isolates. **(A)** There is no significant difference in multiplication rates between single-genotype isolates (*N* = 8) and multiple-genotype isolates (*N* = 26)(mean PMR of 2.7 and 2.5-fold for each respective category, Mann-Whitney test, *P* = 0.9). Isolates were genotyped by PCR of two highly polymorphic loci (details of alleles detected in each isolate are given in Supplementary Table S3). **(B)** There is no significant overall correlation between multiplication rates and numbers of genotypes detected within each isolate (multiplicity of infection, MOI) (Spearman’s rho = − 0.13, *P* = 0.46). **(C)** Data on parasite stages detected in slide examinations at the time of multiplication rate assay initiation were available for 33 isolates. Isolates with any gametocytes detected had a similar multiplication rate distribution (mean = 2.7-fold, *N* = 12) compared to isolates without any detectable gametocytes (mean = 2.5-fold, *N* = 21) (Mann-Whitney test *P* = 0.9).

The possibility that parasite genetic diversity within each isolate might affect the multiplication rates was considered. As a simple measure of the within-isolate genotypic diversity at the time of the multiplication rate assay, highly polymorphic regions of the *P. falciparum msp1* and *msp2* loci were genotyped using a widely-employed PCR method^21^, which showed that each isolate had a different genotypic profile (Supplementary Table S2). Overall, 74% (25 of 34 isolates) contained multiple genotypes, a proportion that was similar among the hospital-based clinical isolates (74%, 17 of 23) and the community-based isolates (73%, 8 of 11). The distribution of multiplication rates was similar for single-genotype isolates (mean PMR = 2.7-fold) and multiple-genotype isolates (mean PMR = 2.5-fold) (Fig. 3A). The isolates each contained up to three different detected genotypes (Supplementary Table S2), but there was no significant correlation between the numbers of genotypes and the multiplication rate of each isolate (Fig. 3B).

As only a small minority of parasites within each cultured isolate normally convert to gametocytes per replication cycle^22^, it was considered that any variation in this would have only a marginal effect on multiplication rates. Gametocyte conversion rates were therefore not measured directly. Examination of Giemsa-stained slides at the point of initiating the multiplication rate assays showed that isolates with detectable gametocytes had a similar distribution of multiplication rates (mean PMR = 2.7-fold) compared to those with no gametocytes detected (mean PMR = 2.5-fold) (Fig. 3C). This does not exclude a possibility that some of the slower growing isolates might have had more early-stage gametocytes at the time of assay, given that these are not morphologically distinguishable.

## Discussion

Intrinsic variation in *P. falciparum*multiplication rates are likely to be clinically relevant. Here, significant differences are shown between multiplication rates in isolates derived from malaria cases attending hospital and from infections sampled from the local community in the same area of The Gambia. The comparison involved establishing continuous parasite cultures from cryopreserved blood samples and performing assays under similar conditions. This showed that parasites from community infections had lower multiplication rates than those from hospital cases, and that there was an overall broad correlation between the in vitro rates and the originally detected in vivo parasitaemia levels. This result is consistent with a hypothesis that intrinsic parasite multiplication rate variation affects likelihood of an infection attaining a high parasitaemia which increases the probability of hospital presentation. However, causality is not yet demonstrated, and it is also possible that variation in the in vivo environment during infection, including differences in host erythrocytes, iron availability and inflammatory immune responses^23–25^, may stimulate parasites to adjust their intrinsic growth rates which then persist in culture.

Previously, a significantly positive correlation between multiplication rates in culture and peripheral blood parasitaemia levels at time of sampling was reported, among isolates from clinical cases from a more highly endemic population in Ghana^13^. It is interesting that parasite multiplication rates in clinical isolates from The Gambia here were lower than seen in the isolates from Ghana, and the initial parasitaemia levels were also lower in Gambian patients. This is consistent with a general positive correlation between parasite multiplication rates in culture and in vivo parasitaemia levels in the sampled subjects, as indicated within each study. Clearly, any correlation is not expected to be absolute, as there will be many other determinants of parasitaemia in the peripheral blood of an individual at the time of sampling, and some community infections with low parasitaemia could be developing towards higher parasitaemia leading to hospitalisation. Parasitaemia levels in the blood of hospital cases here were estimated by microscopy, as was done for the clinical samples from Ghana, while the lower parasitaemia levels in community infections were estimated by quantitative PCR. Future studies may consider estimation of in vivo parasitaemia in all infections by quantitative PCR, and potentially measure plasma levels of parasite histidine-rich protein 2 (HRP2) as another indicator of infection load^1,2^, in populations such as these where parasites do not have deletions of the *hrp2 *gene^26^.

The potential relevance of mixed genotype infection isolates deserves consideration. In a laboratory experimental model, co-infections of two strains of a rodent malaria parasite species in mice tended to be more severe than single-strain infections, although the difference was not clearly attributable to altered multiplication rates^27^. Other studies with the same model have suggested that parasites may increase their virulence during two-strain co-infections^28^. The present study showed no difference in the multiplication rates of *P. falciparum *in cultured isolates with multiple genotypes compared to those with only single genotypes detected. However, it should be noted the multiplication rates measured relate to the whole cultures rather than distinguishing different rates for different component genotypes. Changes in numbers and proportions of genotypes can occur during the first few weeks of culture establishment^29,30^, so the potential of interactions between parasites during in vivo infections is not excluded. As analysis of clinical isolates from Ghana previously showed a trend towards higher multiplication rates in isolates containing single genotypes compared with those having multiple genotypes^13^, overall there is no evidence yet to indicate that human malaria parasites switch to higher multiplication rates in the presence of competitor genotypes.

It remains to be determined how *P. falciparum *evolves or adaptively varies its asexual multiplication rate. Although multiplication rates seen here in the clinical isolates from The Gambia were lower than those from an area of higher endemicity in Ghana, it is unknown whether such rates vary depending on local transmission conditions as has been suggested^31^. Declining endemicity of *P. falciparum*in some areas has been accompanied by observation that most infections have very low parasitaemia levels^15,32^, so it will be important to test a hypothesis that parasites may evolve lower virulence when transmission is reduced by malaria control^31,32^.

One study of parasite gene expression has suggested that there may be increased commitment to parasite sexual stages required to infect mosquitoes when overall transmission rates are lower^33^. The substantial variation in asexual multiplication rates in the present and previous studies is not likely to be explained by varying proportions of parasites undergoing sexual commitment, as these are normally only a small minority of parasites per cycle^13,22^. Consistent with this, the multiplication rate variation in the present study was not significantly associated with presence or absence of gametocytes detected in culture. Future studies could utilise molecular markers of early-stage gametocytes^34 ^to investigate directly whether parasites with particularly low multiplication rates may have unusually high sexual commitment rates at the time of assay. It is recognised that such rates vary over time^22^ but few investigations have been conducted on isolates with low multiplication rates, as experiments on these are difficult to perform.

Clearly, any multiplication rates measured relate to particular culture conditions, and alterations might change these. The use of Albumax for culture media supplementation in this and previous studies using the same methods^12,13^enables standardisation and comparability, while it is recognised that some multiplication rates might differ in media supplemented by serum^35^, just as they might vary depending on donor serum batches. The determinants of relative multiplication rates are also likely to be different if parasites are not tested under exponential growth conditions, for example in competitive co-culture assays at higher parasitaemia levels^4,35,36^.

It is not yet known which phases of the parasite asexual blood-stage cycle contribute most to the significant intrinsic multiplication rate variation among different clinical isolates. It was previously noted that limited variation in the numbers of merozoites within mature schizonts may not have a major effect^13^, but determinants may include merozoite invasion efficiency involving use of alternative receptors, or cell cycle duration^4,14,37,38^. Mutants of many genes affect the growth of long-term culture-adapted parasites^39^, some of which influence tolerance of heat shock for example^40,41^, so further understanding of culture conditions and their effects on understanding natural multiplication rate variation will be important^30^. Overall, an epidemiological as well as experimental perspective on the important parasite trait of variable multiplication potential should guide future studies to understand the genetic and cellular processes responsible.

## Methods

### Parasite sampling from malaria patients and community members

Blood samples were collected in October 2014 from clinical malaria cases attending Basse District Hospital in the Upper River Region of The Gambia, an area that has higher malaria incidence than the rest of the country but lower than previously experienced^17^. Patients were eligible for recruitment into the study if they were aged up to 18 years and had uncomplicated clinical malaria, testing positive for *P. falciparum* malaria by microscopical examination of a Giemsa-stained thick blood smear, and having had no antimalarial treatment within the previous month. Blood samples were also collected between September 2016 and January 2017 in community surveys of villages close to Basse town, as part of other studies on the epidemiology of infection in the area^42,43^. Written informed consent was obtained from parents or other legal guardians of all participating children, and additional assent was received from the children themselves if they were 10 years or older. Venous blood samples (up to 5 ml) were collected into heparinised vacutainer tubes (BD Biosciences, CA, USA), and a proportion of each sample was cryopreserved for later analysis of parasites in culture, adding five times the volume of Glycerolyte 57 reagent (Fenwal Blood Technologies, USA) to a given packed erythrocyte volume after centrifugation. Peripheral blood parasitaemia was estimated by examination of a Giemsa-stained thick blood smear (counting the number of *P. falciparum* parasites per 200 leukocytes and multiplying by the total leukocyte count obtained by automated haematology analysis), or by using a highly sensitive quantitative PCR method^44^ to assay DNA from some of the community samples that had parasitaemia levels too low for accurate estimation by slide examination (< 100 µl^−1^).

## Ethics declaration

Approval for the study was granted by the Joint Ethics Committee of the MRC Gambia Unit and The Gambia Government, and by the Ethics Committee of the London School of Hygiene and Tropical Medicine (reference numbers SCC 1299, SCC 1318, SCC 1476, L2014.40 and L2015.50). All methods were performed in accordance with the relevant guidelines and regulations of the approving institutions.

## Parasite culture

Cryopreserved blood samples were transferred by shipment on dry ice to LSHTM where parasite culture on all samples were performed under controlled conditions within a single laboratory suite. A total of 97 blood samples (35 from hospital cases and 62 from community infections) were thawed from glycerolyte cryopreservation and *P. falciparum* parasites were cultured at 37^o^C using standard methods^45^. No isolates were pre-cultured before the thawing of cryopreserved blood in the laboratory. Briefly, 12% NaCl (0.5 times the sample volume) was added dropwise to each sample while gently agitating the tube to allow mixture, following which the tube was left to stand for 5 min, then 10 times the original volume of 1.6% NaCl was added dropwise, gently agitating to allow mixture. After centrifugation for 5 min at 500 g, the supernatant was removed and cells were resuspended in the same volume of RPMI 1640 medium (Sigma-Aldrich, UK) containing 0.5% Albumax™ II (Thermo Fisher Scientific, UK). Cells were centrifuged again, supernatant removed and the pellet (comprising at least 250 µl for each sample) was resuspended at 3% haematocrit in RPMI 1640 medium supplemented with 0.5% Albumax II, under an atmosphere of 5% O_2_, 5% CO_2_, and 90% N_2_, with orbital shaking of flasks at 50 revolutions per minute. As in previous studies^12,13^, Albumax was chosen for media supplementation rather than serum which is subject to limited donor batch amounts, to enable broader standardisation and comparability across studies. Replacement of the patients’ erythrocytes in the cultures was achieved by dilution with erythrocytes from anonymous donors every few days, erythrocytes being obtained commercially each week (Cambridge Bioscience, UK), so that after 20 days of continuous culture parasites were growing virtually exclusively in erythrocytes from these donors, screened by the provider so that none had sickle cell trait.

Clinical isolates were cultured in parallel in batches of up to 12 in separate flasks at the same time, so that donor erythrocyte sources were the same for all isolates within a batch (Supplementary Table S2). After a few weeks of culture, parasite clinical isolates normally do not contain detectable mutants, which can arise and become common in some isolates after longer periods^8,13,29^. After at least 20 days in culture, a total of 55 isolates (31 from hospital cases and 24 from community infections) had sufficient numbers of parasites to enable initiation of exponential multiplication rate assays, and successful assays were completed on 34 isolates with the required numbers of replicates and quality control parameters as described below.

### Multiplication rate assays

Exponential multiplication rate assays were performed using a method previously described^12^, summarised briefly as follows. Prior to each of the assays which were initiated after more than 20 days of continuous culture of the isolates, fresh blood was procured from three anonymous blood donors in the UK who had not recently taken any antimalarial drugs or travelled to a malaria endemic area, and who did not have sickle-cell trait or other known haemoglobin variants, and erythrocytes were stored at 4^o^C for no more than 2 days then washed immediately before use. Each of the assays for each isolate was performed in triplicate, with erythrocytes from three different donors in separate flasks (erythrocytes commercially provided by Cambridge Bioscience, UK). Asynchronous parasite cultures were diluted to approximately 0.02% parasitaemia within each flask at the start of each assay which was conducted over six days. Every 48 h (day 0, 2, 4, and 6) a 300 µl sample of suspended culture was taken (100 µl pelleted for Giemsa smear, 200 µl for DNA extraction and qPCR), and culture media were replaced at these times. For each parasite isolate, a measure of exponential parasite multiplication rate was derived from all of the datapoints in all of the triplicate cultures over the six-day period, using the following procedure.

Following extraction of DNA, qPCR to measure numbers of *P. falciparum* genome copies was performed using a previously described protocol targeting the highly conserved single locus *Pfs25*gene^12^. Analysis of parasite genome copy numbers per microlitre of extracted DNA at days 0, 2, 4 and 6 of each assay was performed, and quality control excluded any assays with less than 10 genome copy numbers per microlitre. Quality control also removed any single points where a measurement was either lower or more than 20-fold higher than that from the same well two days earlier in the assay, and any outlying points among the biological triplicates that had a greater than two-fold difference from the other replicates on the same day. Assays were retained in the final analysis if there were duplicate or triplicate biological replicate measurements remaining after the quality control steps, and if they showed a coefficient of determination of *r*^2^ > 0.75 for the multiplication rate estimates using all data points. The assay results for 34 isolates (23 from hospital cases and 11 from community infections) passed the criteria. For each assay, an overall parasite multiplication rate (defined as per 48-hour typical replicative cycle time) was calculated with 95% confidence intervals using a standard linear model with GraphPad PRISM.

This overall multiplication rate estimate for each isolate combines all of the data for all replicates over the six-day assay, expressed as a single per 48-hour rate. This rate is derived as a composite from all component parasites within a culture, not distinguishing estimates for different genotypes within a mixed-genotype infection, and not distinguishing between overlapping parasite cycles that co-occur within the asynchronous cultures (the per 48-hour denominator is merely a standardised time that approximates to a typical cycle time and does not imply exact component cycle times of any individual parasites as these are not measured). The *P. falciparum* clone 3D7 was tested in parallel as a control in all assays, consistently showing a multiplication rate of approximately 8.0 fold per 48 h as described previously^12^.

### Parasite genotyping

To test for the presence of single or multiple *P. falciparum* genotypes within each of the cultured isolates at the time of the multiplication rate assays, highly polymorphic marker loci were analysed by PCR. For each isolate, genomic DNA was pooled from the separate time points and replicates in the six-day exponential multiplication rate assay, and alleles of the highly polymorphic repeat sequence regions of the *msp1* and *msp2* loci were discriminated using a nested PCR protocol with visual scoring of different allelic sizes on 1% agarose gels^21^. For each isolate, the different alleles at each locus were enumerated, and the number of alleles at the locus that had the most detected was considered as the genotypic multiplicity (minimum number of haploid parasite genotypes detected).

## Electronic supplementary material

Below is the link to the electronic supplementary material.

Supplementary Material 1

## Data Availability

All data generated or analysed during this study are included in this published article (and its Supplementary Information files).
