# Publisher Correction: Multiplication rate variation of malaria parasites from hospital cases and community infections

**DOI:** 10.1038/s41598-025-90403-7

**Published:** 2025-02-25

**Authors:** Lindsay B. Stewart, Elena Lantero Escolar, James Philpott, Antoine Claessens, Alfred Amambua-Ngwa, David J. Conway

**Affiliations:** 1 Department of Infection Biology, London School of Hygiene and Tropical Medicine, Keppel St, London, WC1E 7HT UK; 2 LPHI, MIVEGEC, INSERM, CNRS, IRD, University of Montpellier, Montpellier, France; 3 MRC Unit The Gambia at London School of Hygiene and Tropical Medicine, Banjul, The Gambia; 4 Department of Infection Biology, Faculty of Infectious and Tropical Diseases, London School of Hygiene and Tropical Medicine, Keppel St, London, WC1E 7HT UK

Correction to: *Scientific Reports* 10.1038/s41598-024-82916-4, published online 03 January 2025

In the original version of this Article, an outdated version of Figure 2 was typeset.

The original Figure 2 and its accompanying legends appear below.

**Fig. 2:**
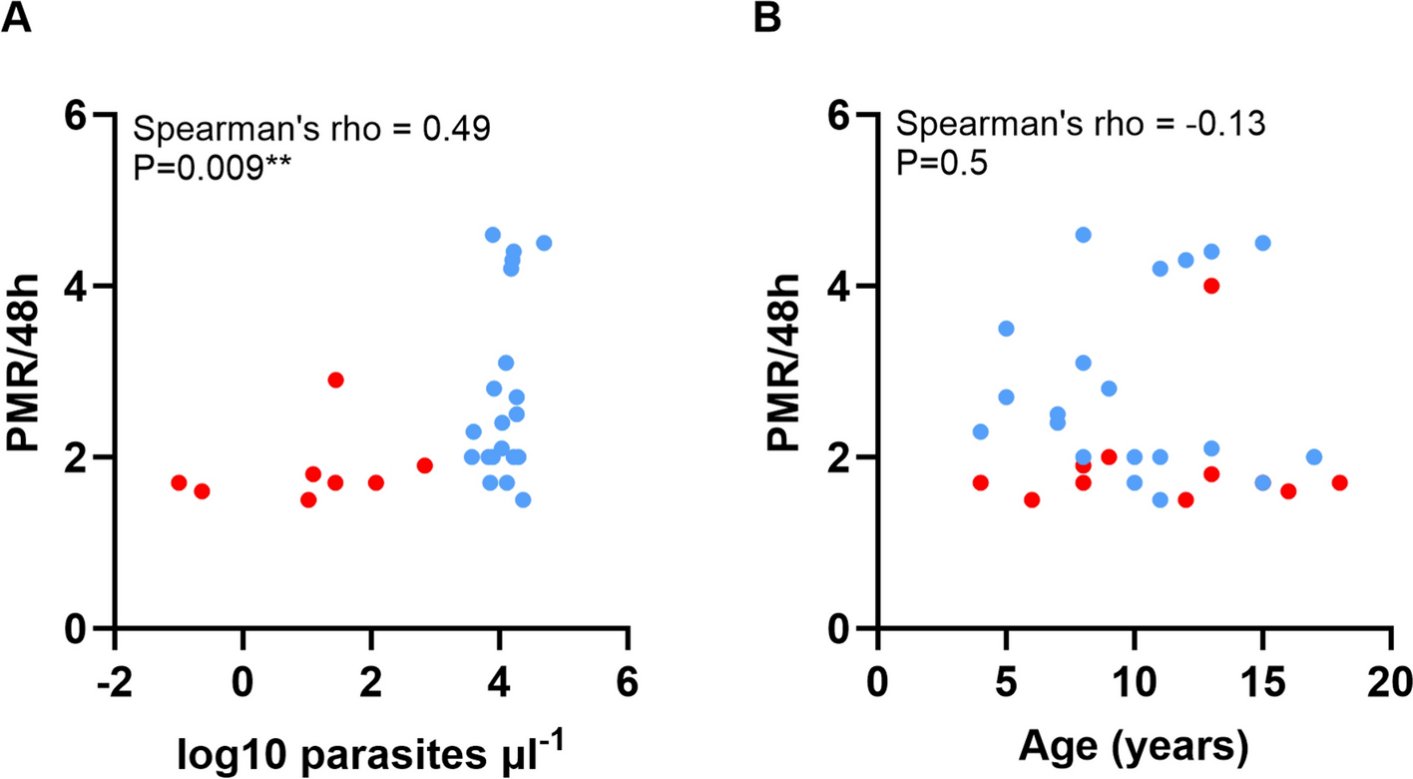
**(A)** Correlation between parasite multiplication rates under exponential growth conditions in culture and the parasitaemia measured in Gambian individuals at time of original blood sampling. Red symbols show community isolates, and blue symbols show hospital isolates (measurements were available for all except six of the 34 isolates as shown in Supplementary Table S1). Analysis of all samples together shows a significant positive correlation (*N* = 28, Spearman’s rho = 0.45, *P* = 0.017). Although there is limited power to test correlations within each of the subgroups, these have slight positive trends (community isolates *N* = 8, rho = 0.54; hospital isolates *N* = 20, rho = 0.06). (**B**) There is no significant correlation between parasite multiplication rates and age of patients (available for all except two of the isolates, *N* = 32, rho = − 0.03, *P* = 0.9).

The original Article has been corrected.

